# Improving healthcare information for young people with ADHD in general practice: a qualitative study

**DOI:** 10.3399/BJGP.2024.0755

**Published:** 2025-07-15

**Authors:** Anna Price, Kieran Becker, Rebecca Gudka, John Headley Ward, Jane R Smith, Faraz Mughal, GJ Melendez-Torres, Emma Pitchforth, Tamsin Newlove-Delgado

**Affiliations:** 1 Faculty of Health and Life Sciences, University of Exeter, Exeter, UK; 2 Department of Psychiatry, University of Cambridge, Cambridge, UK; 3 School of Medicine, Keele University, Keele, UK

**Keywords:** ADHD, digital resources, healthcare information, primary health care, qualitative research

## Abstract

**Background:**

Attention deficit hyperactivity disorder (ADHD) is a common neurodevelopmental disorder that can have poor long-term outcomes when unmanaged. Young people aged 16–25 years with ADHD are often unable to access specialist health care as recommended by UK guidelines because of gaps in services, poor transitional support between child and adult services, and long waiting lists. Healthcare information, which is important for condition management, may help mitigate service gaps and support thriving in people with ADHD; however, little is known about provision via primary care.

**Aim:**

To investigate experiences of information provision supporting management of young people with ADHD in general practice and explore the potential of digital resources.

**Design and setting:**

This qualitative study comprised interviews with young people with ADHD, their supporters, and primary healthcare professionals from sites across England.

**Method:**

Participants were recruited from five purposively sampled general practices, varying by local area characteristics. Semi-structured interviews included questions about information provision, healthcare information needs, and digital resources. Themes were generated using reflexive thematic analysis, within a critical realist framework.

**Results:**

In total, 20 participants were recruited (11 healthcare professionals and nine people with lived experience). Four themes were generated: lack of ADHD-specific resources, supporting patients with condition management, dedicated resources for clinicians, and digital resources enhancing care*.*

**Conclusion:**

People with lived experience and healthcare professionals want better healthcare information about ADHD in general practice, including co-produced resources to support understanding and self-management. Digital resources represent a potentially cost-effective and accessible solution that is currently underutilised.

## How this fits in

Healthcare information can inform clinical practice and support patients to positively manage long-term conditions; however, accessing curated and trustworthy information on attention deficit hyperactivity disorder (ADHD) can be challenging for patients, especially given ongoing controversies around the diagnosis. This qualitative study investigates lived experience and clinical perspectives on provision of healthcare information via primary care for people with ADHD and explores the potential for digital resources to address existing gaps. Our findings indicate that there are insufficient ADHD-specific, evidence-based resources available via primary care and demonstrate enthusiasm for accessible digital resources to inform and support management of ADHD. People with ADHD and healthcare professionals recommended that digital resources should be co-developed, to ensure they are engaging and accessible.

## Introduction

Attention deficit hyperactivity disorder (ADHD) is a neurodevelopmental disorder characterised by hyperactivity, impulsivity, and/or inattention,^1^ with an estimated prevalence of 2%–7% in children and adolescents,^2–5^ and 3% in adults.^6,7^ Untreated ADHD can lead to many adverse outcomes and have a negative impact on multiple life domains.^8–11^ Young people aged 16–25 years with ADHD are particularly vulnerable, facing multiple transitions such as leaving home or starting work, and increased risks of developing comorbid mental and physical health conditions compared with the general population.^6,11–13^ ADHD can be effectively treated with medication,^10,14–17^ and non-medical interventions such as tailored behavioural and cognitive therapies are important for positive condition management.^18^ However, in the UK, access to ADHD health care is limited because of stretched services, long waiting lists, unsupported transitions between child and adult services, and complex care pathways.^19–21^ These systemic problems often leave primary healthcare professionals (HCPs) supporting patients without adequate ADHD-specific resources, support, or guidance.^22–24^

Provision of evidence-based healthcare information in accessible formats can help young people with ADHD to navigate care pathways, understand their condition, anticipate changes in symptoms as they age, and manage health risks.^25–28^ It can also inform clinical practice.^29^ UK guidelines recommend that people with ADHD and their supporters receive structured discussions to improve understanding of ADHD, and signposting to sources of information, such as charities and websites.^30,31^ However, most young people with ADHD experience limited or no access to this element of health care, with less than a third of specialist services providing the recommended range of treatments.^31,32^ When accessible, treatments typically focus on medication, without providing adjunctive psychoeducation or key healthcare information.^20^ For young people this gap can be especially damaging because of the varied ways ADHD presents at different life-stages, which makes understanding symptoms challenging, and the high levels of misinformation online.^18,25,28,33,34^

Digital technologies, including interventions delivered via primary care, represent a potentially cost-effective and accessible way of providing healthcare information about ADHD to young people and their supporters, while placing minimal demands on HCPs.^26,35^ In the context of increasing and competing demands on the NHS, digital interventions, including apps, are providing scalable ways of enhancing mental health care.^36–38^ Similar interventions could help address unmet need in young people with ADHD.^21^ Primary HCPs are often the first and most consistent point of contact for young people with ADHD,^19,23^ so can provide access to trusted, curated, and evidence-based resources. However, little is known about provision of ADHD healthcare information and digital resources in this setting. This study aimed to explore the perspectives of patients, supporters, and HCPs on current delivery of healthcare information for young people (aged 16–25 years) with ADHD via primary care, and on the potential role of digital interventions.

## Method

### Participants and recruitment

The Managing ADHD in Primary care (MAP) study used mixed methods to explore primary care provision for young people with ADHD in England.^24,39^ Delivery was guided throughout by regular meetings with two research advisory groups (RAGs). One was made up of people with lived experience of ADHD and another of HCPs. The aim was to recruit 10–15 young people with ADHD and their supporters and 10–15 primary care professionals located across a range of locations (three to six) to reflect diverse experiences. Potential study sites were identified via responses to the MAP national survey where participants had expressed an interest in further research engagement,^19^ and purposively sampled to reflect varied local area characteristics (rural/urban setting, ethnicity, and deprivation). Participants were recruited through sites, or via survey responders located near sites. Participants had to be people with lived experience of ADHD (young people aged 16–25 years with ADHD or a supporter) or primary HCPs. All participants lived or worked in England. The MAP protocol provides further details of the recruitment strategy and public and patient involvement and engagement.^39^

### Data collection

Data were collected via semi-structured interviews (approximately 60 min) using topic guides; see (Supplementary Information S1). This research focused on data exploring information resource use and the potential for digital solutions to support management of ADHD in primary care (Box 1). Findings from other topics are reported elsewhere.^24^ Topics reflected lived experience priorities highlighted during engagement work.^25,28^ Question wording was developed with MAP study RAGs, made up of people with lived experience and HCPs.

Supplementary Information

**Box 1.** Summary of topics used to generate data, by participant group

Healthcare information, resource use, and exploring digital solutions to help manage **ADHD**	
**Young people and supporters** This section asks about resources to provide information to help manage and self-manage the health of people with ADHD.	**Healthcare professionals and commissioners** This section asks about information resources to help manage the health care of patients with ADHD. The focus is on resources you use to aid your practice, and resources you share with patients to help them understand and self-manage their condition.
Please tell us about ADHD healthcare resources you have been given by your practice.	Please tell us about resources you use to help manage the needs of patients with ADHD.
What other resources do you use to help manage health and wellbeing with ADHD?	Please tell us about resources you signpost patients with ADHD to.
In an ideal world, what resources would be available from your GP to help manage ADHD?	In an ideal world, what kind of resources would you like to be available in primary care to help manage ADHD patient needs?
If someone told you about a fab digital tool for people with ADHD, what would it be like and what would it do?	If someone told you about a fab digital tool to aid treating patients with ADHD, what would it be like and what would it do?
Please tell us about tools for managing other health conditions that could be adapted for ADHD.	Please tell us about tools for managing other conditions that could be adapted for use with ADHD.
Do you have anything to add?	Do you have anything to add?

ADHD = attention deficit hyperactivity disorder.

Interviews were conducted flexibly to suit participant engagement needs and completed via Microsoft Teams or telephone between March and June 2023, with informed consent. Recordings were transcribed verbatim, with data anonymised and stored on a regulation-compliant secure server.

### Data analysis

A reflexive thematic analysis of data related to information provision and digital solutions was conducted to generate themes, following methodology as described by Braun and Clarke^40^ and operationalised by Byrne.^41^ Analyses were underpinned by a critical realist perspective, which is appropriate for research aiming to address identified gaps in knowledge about health care in practice, and how it could be improved.^42^ This theoretical stance incorporated consideration of the context of the data, and the influence of researcher and stakeholder perspectives, providing a nuanced interpretation of meaning. Data were managed using NVivo (version 14).

Two researchers (the first and second authors) immersed themselves in the data by reading all transcripts. Line-by-line coding was undertaken. An initial inductive framework was created from sections of the topic guides, then refined deductively from the data, and in consultation with the study team (the third and fourth authors and the senior author). Themes and sub-themes were generated. Coders documented and reflected on personal perspectives throughout via group discussions and journals. Column summaries were created for a framework matrix, then organised into themes and sub-themes. Generated themes were iteratively refined in consultation with RAG members and the co-authors. Data relevant to improving practice were collated and transformed into an infographic sharing recommendations for practice. See the MAP protocol for further detail.^39^

## Results

### Sample

Following RAG consultation, five primary care practice sites were chosen to reflect varied local area characteristics and a variety of reported care contexts (shared-care prescribing and specialist service availability). These included a university-linked practice, and practices with and without specialisms in substance misuse and homelessness. In total, 20 participants (11 HCPs and nine people with lived experience) were recruited from five diverse primary care practice sites located across five NHS England regions. See Supplementary Table S1 for detailed site characteristics. Table 1 details participant characteristics.

**Table 1.** Unique identifier, role, and other characteristics of participants

Site	Unique identifier	Role (age range in years)	Sex, ethnicity (self-described)	Additional information (provided at interview)
1	PracticeManager-1	Practice manager	Female, White British	Managing partner
1	YoungPerson-1	A young adult with ADHD (18–25 years)^a^	Male, White British	Student
1	YoungPerson-2	A young adult with ADHD (18–25 years)^a^	Female, White British	Graduate
2	GP-1	GP^a^	Female, White British	Commissioning experience, ADHD in family
2	GP-2	GP	Male, White British	ADHD in family
2	PracticeManager-2	Practice manager	Female, White British	Possible undiagnosed ADHD, ADHD in family
2	YoungPerson-3	A young adult with ADHD (18–25 years)	Female, White	Mother
2	Supporter-1	A supporter of a young adult with ADHD (18–25 years)	Male, White British	Grandfather
3	YoungPerson-4	A young person with ADHD (16 or 17 years)^a^	Male, White British	Apprentice
3	Supporter-2	A supporter of a young person with ADHD (16 or 17 years)^a^	Female, White British	Mother
3	GP-3	GP	Female, British Mixed	Commissioning experience, ADHD in family
3	PracticeManager-3	Practice manager	Female, White British	—
4	PracticeManager-4	Practice manager	Female, White	—
4	WellbeingWorker	Wellbeing worker	Female, White other	Neurodiverse
4	GP-4	GP	Male, Irish	—
4	YoungPerson-5	A young adult with ADHD (18–25 years)	Female, White British	Student
5	Supporter-3	A supporter of a young adult with ADHD (18–25 years)^a^	Female, White British	Mother
5	YoungPerson-6	A young adult with ADHD (18–25 years)^a^	Female, White British	On a break from university
5	PracticeManager-5	Practice manager	Female, British Pakistani	—
5	GP-5	GP	Male, British Pakistani	Commissioning experience

a *Participant recruited via Managing young people with ADHD in Primary care study (instead of via site) from location within same local area as practice research site. ADHD = attention deficit hyperactivity disorder.*

### Findings

Four themes were generated regarding provision of healthcare information for young people with ADHD via primary care: lack of ADHD-specific resources, supporting patients with condition management, dedicated resources for clinicians, and digital resources enhancing care. These are summarised in Box 2 and described below.

**Box 2.** Summary of themes

**Themes** Sub-themes	**Summary**
**Lack of ADHD-specific resources** Lack of ADHD-specific healthcare information Variable signposting to resources	ADHD-specific healthcare information is not usually shared with patients by GPs. While a few GPs are aware of ADHD-specific resources, most signpost to generic websites. Patients are often left to seek information via informal routes, which can be unhelpful.
**Supporting patients with condition management** Information increasing understanding of ADHD and care pathways Resources and tools to aid self-management Accessible resources, co-developed with people with ADHD	Information for people with ADHD needs to increase patient and supporter understanding of ADHD and inform navigation of care pathways. Resources and tools to support positive condition management would be helpful. Content needs to be developed alongside people with lived experience and include relatable stories.
**Dedicated resources for clinicians** Improving understanding of ADHD Guiding clinical practice	Primary care professionals need resources to help them understand care pathways, and how to support the needs of young people with ADHD. When supporting patients with ADHD, primary care professionals want and need better-quality resources to guide their clinical practice.
**Digital resources enhancing care** Advantages, including improving access Trusted digital content Risks of exclusion Importance of non-digital	Digital resources could improve care by providing tailored healthcare information and condition management support via primary care. Providing trustworthy digital resources would reduce risks of online misinformation. Some people may struggle to access digital resources, and non-digital options remain important.

ADHD = attention deficit hyperactivity disorder.

### Themes and sub-themes

#### Lack of ADHD-specific resources

##### Lack of ADHD-specific healthcare information

Many participants with lived experience reported a lack of health information resources designed with young people with ADHD in mind. Most participants with lived experience reported receiving *‘not a dot’* (Supporter-2) of ADHD-specific healthcare information from their primary care providers:

*‘They had the more generic mental health, but nothing ADHD-specific*.*‘* (YoungPerson-2)

This lack of information was compared with that for other long-term conditions by one participant with lived experience where trusted healthcare resources were perceived as being readily accessible:

*‘When you get diagnosed with asthma, there’s all sorts of resources available. You get leaflets and all sorts of things … I have never seen any information at all in the surgery that we go to … about ADHD, nothing.’* (Supporter-3)

##### Variable signposting to resources

When asked about experiences of signposting to trusted resources via primary care, most participants with lived experience reported that they had not received any, and several stated their *‘understanding of ADHD has come from* [their] *own research.’* (YoungPerson-5)

Frustrations about this lack of signposting, and implications in relation to health inequalities, were summarised by a young person:

*‘If I want to find out something about ADHD, I primarily use either medical webpages that I trust, or I read research. But I’m also aware that that’s not an accessible option for most people … it’s kind of annoying that I have to do that, because there isn’t anything else that I trust.’* (YoungPerson-1)

Another young person stated:

*‘I am not sure. I know they have some resources on the website … Mostly, I don’t have much engagement with my GP about neurodiversity in general.’* (YoungPerson-6)

Despite reported frustrations, several participants shared details of resources that they had identified independently and found useful, including *‘YouTube channels’* (YoungPerson-5), *‘podcasts’* (YoungPerson-6), and the *‘ADHD Foundation’* (Supporter-2).

By contrast, many HCPs reported routine signposting practices. However, approaches used and specificity of resources shared varied. As one HCP stated, *‘Different clinicians are aware of* [different] *things’* (PracticeManager-1). Several GPs mentioned signposting to websites such as *‘nhs.uk’* (GP-5) or relying on clinic letters:

*‘In terms of online resources for patients, I tend to find that the clinic letters that come with patients tend to have a long list at the end of those online resources. So, I personally don’t feel I need to point them in those directions because … I think most students will be aware of what is available.’* (GP-4)

Another GP stated:

*‘So, we use the NHS website … we use patient.co.uk … so they will have some ADHD advice and guidance. And it’s fairly robust, but there is an opportunity for people to go away and trawl the internet to find information that they find useful.’* (GP-5)

Meanwhile, some described ADHD-specific resources including podcasts, videos, and books:

*‘I direct them towards the ADHD Foundation to see if any of that is of additional support to them, I will print off or text them information from NHS sources on ADHD.’* (GP-1)

### Supporting patients with condition management

#### Information increasing understanding of ADHD and care pathways

Many patients and supporters said having information to increase understanding of living with ADHD would have helped them recognise when they needed help, and better understand ADHD needs:

*‘When I first got diagnosed with ADHD, it was sort of just "Oh, right, you’ve got ADHD", sent you on your way … it would have been nice to have something to look at where it tells me about it. And I know everyone with ADHD is different, but just some understanding of what’s going on in my head.’* (YoungPerson-4)

Participants said resources to help supporters understand ADHD were also crucial as *‘parent’s understanding has also a massive impact’* (PracticeManager-3). One supporter explained why clearer information on care pathways was needed:

*‘From a family perspective as well, more information would be really, really helpful because … we end up scrabbling around … and often it will be me that picks meds up etc., so support materials would actually be really helpful*.*’* (Supporter-3)

#### Resources and tools to aid self-management

Many young people mentioned wanting more knowledge and understanding about topics important to them, such as living with *‘comorbidities’* (YoungPerson-6), *‘female presentations’* (YoungPerson-5), and *‘how ADHD can impact your mental health, and your relationships’* (YoungPerson-1), implying a need for psychoeducation to support long-term condition management. One young person stated:

*‘Information about things like how to sleep well; how to make sure that you can keep habits. Because with ADHD, you have to take a very specific approach … Because I feel like, if you have ADHD, it’s a lot harder to manage your health.’* (YoungPerson-1)

Participants across stakeholder groups highlighted a need for condition management resources. Suggestions included *‘guided self-help, CBT* [cognitive behavioural therapy]*‘* (GP-3), *‘planners, reminders’* (YoungPerson-3), and information to *‘enable you to figure out the best way for you to go about tasks’* (Supporter-3). Participants consistently emphasised the need for tailored self-management tools that would *‘work for ADHD, rather than being very general’* (YoungPerson-1):

*‘To have more specific support relating how those symptoms might manifest in different situations and how you might want to manage it would probably be quite useful … It is quite difficult to do without anyone else helping you.’* (YoungPerson-6)

A wellbeing worker from a university practice said tools for managing procrastination were a priority:

*‘And most of the time, even people who may have had that* [ADHD] *diagnosis for a while, would still be coming in with the same question, "How do I work with procrastination?’’’* (WellbeingWorker)

#### Accessible resources, co-developed with people with ADHD

Several HCPs and participants with lived experience emphasised that resources needed to be engaging and accessible to people with ADHD. One HCP suggested resources should:

*‘... be co-produced with people with ADHD so that it looked right in terms of the chunking of the information ... making that document as accessible as possible*.*‘* (GP-3)*.*

Another GP stated:

*‘I think sometimes good-quality narrative accounts can often be very helpful … saying here are the issues I grappled with, and this is what I found helpful … because sometimes these documents can be very, very dry and it can be very difficult to actually know what something means to a patient’s experience.’* (GP-4)

Several young people reported finding peer stories and neurodiverse-led resources helpful:

*‘If there was something where it’s stories from people with ADHD, it will help … I was talking to one of the other lads with ADHD, and when we started talking, he went through a lot of the same stuff I did when I was growing up with it, so it’s a lot more relatable if you hear about it from somebody with ADHD.’* (YoungPerson-4)

*‘The best ones, honestly, are … led by people who have ADHD themselves. There’s nothing that can beat that*.*‘* (YoungPerson-2)

One young person suggested that advice on self-management should not come from an *‘outside perspective’* (YoungPerson-5).

### Dedicated resources for clinicians

#### Improving understanding of ADHD

Participants across stakeholder groups outlined the need for resources to improve ADHD awareness in general practice, including what it is like living with this condition:

*‘more training on neurodiversity is absolutely crucial, and I think it is coming … Everybody needs to know about it so I think it’s disingenuous having just one person knowing about it.’* (GP-3)

*‘One thing I think could be really useful is if there were videos or information with metaphors or analogies about what the internal experience of ADHD is like for neurotypical people.’* (YoungPerson-5)

*‘If they don’t know anything about ADHD, and they have to go look it up, it’s less helpful … if they knew just the basics, and they were like, “Okay, so first things first, you refer to this, and then you do this.” Or “Okay, you need to talk to that person, who specialises.” ... Because when I ring them up about anything, you hear that hesitation on the other end of the line, where they’re like, “I’m going to have to go talk to someone about where to send you."’* (YoungPerson-2)

#### Guiding clinical practice

One GP stated:

*‘So yes, number one I guess is the understanding as a GP. My training in ADHD, my access to resources that explain to me how to diagnose and provide initial treatment and management is limited. It’s improving … but it’s still got a long way to go.’* (GP-2)

Some GPs expressed confidence in available resources for informing their management of ADHD:

*‘Yes. I mean, I would personally make use of that shared-care protocol that I mentioned because it gives, I think, fairly clear advice from a GP point of view.’* (GP-4)

However, others said protocols could be unclear, and observed that when specific questions arose, for example, around managing ADHD medication, they *‘don’t really have anybody who* [they] *can go and talk to, to ask those questions’* (GP-2). This GP went on to say:

*‘Perhaps we don’t have adequate training in understanding how to use the medication effectively. Not that we’d necessarily expect to be changing doses but understanding the significant side effects that some of these medications have and the way that people learn to use them to the best effect … things like that.’* (GP-2)

### Digital resources enhancing care

#### Advantages, including improving access

Many stakeholders saw advantages in using digital resources to communicate healthcare information about ADHD, including making information easier to access and update:

*‘I think … a digital tool is always helpful because then it’s very easily accessible … it can be updated, or the information can be nested, and you can follow more complex pathways … whereas the paper one is a bit unidimensional.’* (GP-4)

Many discussed the importance of tailoring healthcare information to be accessible and relevant for young people with ADHD. Noted advantages were that digital resources could be available 24/7, and via multiple formats (for example, video, text, or games), which is important for people with difficulties regulating attention:

*‘If there is a tool that does assist somebody with ADHD, a quick response. I think the most frustrating thing for people is needing help and not being able to get it quickly.’* (PracticeManager-3)

*‘I think videos are a great way because a lot of kids don’t want to sit and read, but they’ll listen to somebody, say, like, or somebody that has lived* [experience] *… would make a massive difference.’* (Supporter-2)

Participants outlined other ways in which digital technology could improve care, including through delivering *‘virtual clinics’* (GP-1), and supporting ordering of medication and booking appointments. One discussed how digital assessments might support clinical conversations:

*‘I think the digital platform could be a really useful way forward, because if there were one health professionals accepted as a recognised and appropriate tool where a person could come in and say "I’ve done this assessment on this tool, here’s my assessment, scores", that would be a helpful introduction, wouldn’t it?’* (PracticeManager-4)

#### Trusted digital content

Both HCPs and participants with lived experience emphasised the importance of curated and trustworthy digital content. Several HCPs said digital resources needed to be *‘evidence-based’* (PracticeManager-1) and easily available:

*‘I mean it would be useful to have something* [digital] *somewhere that is reliable and well curated so the information that we are looking at is as trustworthy as it can get and that contains information that’s more hard clinical.’* (GP-4)

Participants with lived experience said they would value access to digital resources provided via a trusted source such as their GP:

*‘I think ideal would be, basically, imagine you had a web page, that just had a bunch of links on it. And it was like, “Here’s everything. This is the link to the support group; this is the link to the online resources; this is a web page that’s all about ADHD, or something.” Just one place, basically, where they’re like, “Here’s the ADHD things” So, you don’t have to search so many different places to get it.’* (YoungPerson-2)

Participants with lived experience discussed risks of misinformation, and reflected on challenges faced by individuals left to navigate online information without support:

*‘... social media … sometimes isn’t the best place to go. I mean, that’s me personally, but I think there’s a lot of misinformation on the internet … Because social media is mostly young people. And if they get the wrong information, they’re not old enough to understand it and it’s not the right information.’* (YoungPerson-4)

#### Risk of exclusion

However, it was noted that digital resources could create inequalities. One grandparent explained that digital help might not be accessible to him:

*‘I’m obviously not clued up on the technology side of things … I can access an iPad and things like that but I’m very, very limited.’* (Supporter-1)

A young person with ADHD discussed how reliance on digital support could unintentionally exclude people from disadvantaged backgrounds:

*‘Now, if you’re from a poverty family who can’t travel, and haven’t always got access to the internet, there’s nothing for the parent or the child, face to face or even trying to get onto a laptop in a library or whatever, there’s nothing.’* (YoungPerson-4)

Some participants outlined problems with existing digital resources. One noted they can be poorly designed and costly:

*‘I had to do an assignment for uni, that was evaluating a bunch of apps to help with stress … They were alright, but they were all badly designed, in terms of their actual usability. They tended to be quite laggy. And if they weren’t laggy, they were behind a massive paywall.’* (YoungPerson-1)

#### Importance of non-digital

Several participants noted the importance of providing non-digital options, so that digital resources became part of *‘a multitude of resources in different forms … so that people who focus better on different things have an option that works for them’* (YoungPerson-5)*.* Key examples included conversations with staff with expertise in neurodiversity and printed materials:

*‘… what is the most helpful thing is the actual coaching, and also the opportunity for people to say what it’s like.’* (WellbeingWorker)

*‘Obviously, GP services have got a lot to do, which is why I think, a leaflet, if it was like just on the reception and …* [they say] *"Can you have a look through this while you are waiting?" I think that could be really cool.’* (YoungPerson-5)

### Practice recommendations

**Figure 1.**
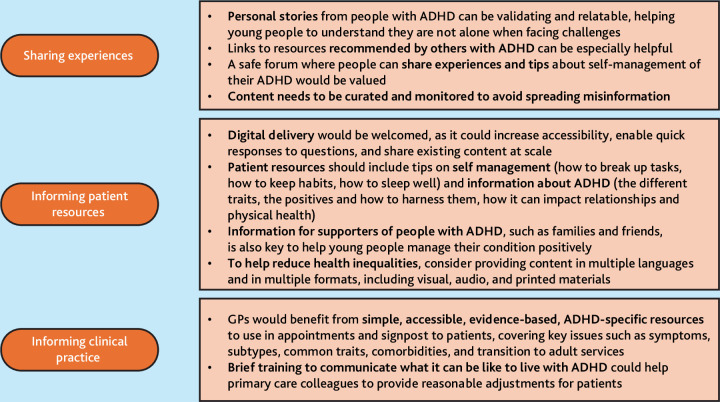
Key recommendations for providing healthcare information to support management of ADHD via primary care. ADHD = attention deficit hyperactivity disorder.

Practice recommendations are presented in Figure 1.

## Discussion

### Summary

This research builds on findings about information provision for young people with ADHD in secondary services,^25,28^ providing novel data about the primary care context. Our findings tie in with cross-sectional survey and qualitative research, further evidencing geographic variations in NHS support for young people with ADHD.^19,20,25,28,43^ The data highlight a lack of accessible healthcare information about ADHD and care pathways, despite UK guidance recommending provision.^31,44^ These gaps are having a negative impact on healthcare transitions and management of ADHD in young people. Our research also provides a nuanced exploration from multiple perspectives of the potential for digital technology to address unmet needs in ADHD health care, including through tools that support self-management.

Findings highlight an opportunity for trusted and curated digital resources, delivered via primary care, to guide clinical practice and support positive management of ADHD. Our recent systematic review provides preliminary evidence of the feasibility and acceptability of digital healthcare information interventions for young people with ADHD, especially those that are co-produced.^45^ However, no such tools currently meet NHS England standards. This novel research highlights opportunities for stakeholders to work together to co-design and evaluate digital healthcare information interventions, designed for implementation within primary care pathways. Such tools would have the potential to reduce GP burden, while better meeting the needs of patients with ADHD.

### Strengths and limitations

This study benefits from a diverse range of perspectives, including practice managers, GPs, and young people with ADHD. However, given service pressures, the HCPs we recruited may have been more likely to have existing knowledge of ADHD and we may have under-represented perspectives of HCPs without an interest in ADHD. People with lived experience may have been more likely to engage if they had experienced challenges accessing health care, meaning some positive experiences may not be reflected in these findings. Although every participant with lived experience self-described as White British, two RAG members with ADHD (who are from different ethnic backgrounds) contributed to team discussions about ethnicity. This informed data syntheses and has influenced future research plans. Purposive sampling of study sites to include rural and urban practices, and those situated in areas of high and low deprivation, helped the study reflect local variations. However, given the thousands of practices in England and heterogeneity of communities, data from participants at five sites are unlikely to have reached saturation in relation to the breadth of experiences and perspectives.

### Comparison with existing literature

This research highlights the poor availability of resources to inform and support management of ADHD in young people, especially compared with other long-term conditions such as asthma.^46^ Failures to provide high-quality healthcare information can leave patients relying on alternative informal sources, making them vulnerable to misinformation and potentially causing unintended harm.^33,34^ HCPs reported signposting as part of routine care, but, in line with previous findings, few reported providing ADHD-specific information.^25,28^ Some also observed that standard practices, such as adding links to referral letters or sharing verbal recommendations, might not represent optimal communication for a group who struggle with memory and focus.^18^ Prior research emphasises the importance of communicating via varied and accessible formats when supporting young people with ADHD.^47^ Based on these findings, we recommend collating existing resources,^48,49^ and co-developing a digital suite of evidence-based ADHD-specific resources, tailored for young people, and suitable for delivery via primary care.^50,51^

This research raises and answers some questions about optimising resources for primary HCPs.^22,52^ GPs need improved guidance on care pathways and resources to better understand ADHD in young people.^23^ It is possible that improved shared-care protocols, with advice covering topics such as medication and monitoring, might help, especially given difficulties GPs face seeking specialist clinical advice.^21,23^ Web-based training has been shown to have a positive impact on practice;^53,54^ however, given time constraints, curated digital resources delivered to GPs at the point of need (for example, before/during a consultation) may be more practical for implementation at scale.

Digital technologies, including interventions for patients, and clinical guidance integrated into existing IT systems for HCPs, are potentially powerful tools for providing healthcare information and self-management tools within stretched primary care services.^55,56^ Digital resources to support young people’s mental health are already being recommended for use in the NHS.^36^ However, further research is needed to develop similar interventions for young people with ADHD.^48^ Some aspects of digitally enhanced usual care for ADHD have been explored, including tools aiming to improve attention and increase medication adherence,^49^ and smartphone apps delivering self-management tools and psychoeducation.^57^ However, most interventions lack a robust evidence base, fail to include stories of those with lived experience, or are only available locally,^57–59^ which means they do not meet many desired characteristics highlighted in this research. Our findings show that stakeholders want curated digital resources, shared via a trustworthy source such as the NHS.^60^ Further work is needed to develop such resources and explore system-wide implementation.

### Implications for research and practice

There is an urgent need to improve primary care management of ADHD by providing trusted healthcare information and condition management resources to HCPs and patients. Patient resources need to be co-developed, provide ADHD and life-stage specific information, incorporate self-management tools and psychoeducational content, include patients’ stories, and be available via multiple formats that are accessible to ‘ADHD-brains’. Digital technologies, including healthcare apps for patients and treatment guidance for clinicians, have the potential to deliver such resources cost-effectively and at scale. Future research needs to integrate the perspectives of multiple stakeholders and address risks of digital exclusion.^61^ Co-developing and delivering such resources could reduce health inequalities, enhance the health and wellbeing of young people with ADHD, and improve job satisfaction within general practice. A national strategy, with a coordinated approach to implementation, would be important to ensure comparable levels of care and information nationally. The newly formed NHS England ADHD Taskforce represents a unique opportunity to deliver in this area.^62^
