# Supplementary material for: Improving healthcare information for young people with ADHD in general practice: a qualitative study
Source: Br J Gen Pract. 2025 Jul 15;75(758):e586–96. doi: 10.3399/BJGP.2024.0755 (PMC12723502; doi:10.3399/BJGP.2024.0755)
Supplement: Supplementary file 1 — Supplementary Information [file bjgp-2024-0755_suppl1.pdf]

Supplementary Materials

Contents

Supplementary Information S1. Topic Guides..1
Table S1 with summary of research site characteristics.............................8
References 9

Supplementary Information S1. Topic Guides

Interview Topic Guide - WP2

(Health professionals and providers)

Study title: Managing young people with ADHD in primary care (MAP) study
Chief Investigator: Dr Anna Price a.price@exeter.ac.uk. Telephone 01392 726026

Please note this topic guide is for researchers only. This will be used to prompt questions during the interview. This is not a questionnaire. This topic guide will be adapted and refined following findings from WP1, and in consultation with MAP study research advisory groups.

This interview aims to explore your perspectives and experiences in relation to providing services and support for young people (aged 16-25) and adults with attention deficit hyperactivity disorder (ADHD) in primary care (e.g., GP practice or local surgery). There will be a particular focus on factors that have made it easier and those that have made it harder to provide accessible and appropriate care. Also, on any recommendations you may have to improve access to primary care for people with ADHD in the future.

Demographics

This section asks for brief background information about your role and experience.

- Please tell us briefly about your primary care role? *(GP, specialist nurse, PCN manager, pharmacist. Any other roles such as commissioning. How long in role?)*
- What is the postcode of your workplace? *(GP practice)*
- What is your sex (gender)? *(Male, Female, Non-binary / third gender, prefer not to say)*
- Which ethnic group best describes you?
    a. *Asian, Asian British, Asian Welsh*
    b. *Black, Black British, Black Welsh, Caribbean or African*
    c. *Mixed or Multiple*
    d. *White*
    e. *Other ethnic group*
    f. *Prefer not to say*
- Do you have any professional/personal interests in neurodevelopmental disorders/mental health that inform your work?

Research topic guide

1. How people with ADHD access primary care

This section asks about your experiences of the ways young people and adults with ADHD access primary healthcare.

a. How do patients with ADHD **make and attend appointments** with your practice? What are the barriers? What makes it easier? *(Supporters/ Moving home/ University/ Covid-19/ Face-to-face/online/phone)*
b. Is there anything that your service does to make it easier for patients with ADHD to **access** care and support?
c. Are you aware of any barriers for particular groups of patients, and do you have anything in place to mitigate these?
d. When patients with ADHD move GP practice (e.g., moving to university, moving home) what usually happens in terms of their access to primary care?
e. In general, what can primary care providers do to make it easier for young people with ADHD to attend appointments and get the help they need when they need it?

2. Support for primary care providers working with young people and adults with ADHD.

This section asks for your perspective on how local providers and the wider healthcare system can support primary care professionals to provide healthcare for patients with ADHD.

a. What current challenges do you face in providing primary care to patients with ADHD within the existing system? Are there any measures that could be implemented to make this easier? *(at a practice, PCN or national level)*
b. In general, what systems/structural changes could be made to help you and your service to provide better care for people with ADHD? *(Financial incentives, training, staffing, information resources, decision making tools, care pathways, technology, digital resources)*

3. Providing care and support for people with ADHD through primary care

This section asks about your experiences of providing healthcare and support for young people and adults with ADHD, including barriers faced and what makes it easier. Also about differences in experience/process when providing care for patients via NHS, right to choose, or private diagnosis routes.

Patients

a. Please tell us about patients with ADHD at your practice/in your primary care network (PCN)? *(How many there are/ Roughly how many might you see in a year/ How often do you see them).*
b. Can you describe their characteristics? *(gender, life circumstances, age range)*

Prescribing

c. Do staff at your practice have access to an 'advice and guidance' service from AMHS professionals with specialist knowledge of adult ADHD? *What sort of support would you find useful from AMHS?*
d. Does your practice have shared care protocols/agreements in place with an AMHS to enable prescribing of adult ADHD medications through primary care for patients? (**NHS/Private diagnosis**)

e. If relevant, how does your practice manage prescribing adult ADHD medications for patients? (**NHS/Private diagnosis**)
f. What do you or your practice undertake to do as part of shared care, to enable safe prescribing of ADHD medication?
g. Please tell us about differences in your experiences of providing ADHD care for patients with different circumstances *(NHS/ right to choose/ private. Diagnosis from another country. Transition. Moving house)*

Other ADHD healthcare (non-pharmacological)

h. Please tell us about any non-pharmacological support your practice provides for patients with ADHD *(Social prescribing/ mental health/ support groups)*
i. Please tell us about your experiences of managing treatment for ADHD when patients go through change. *(transition to AMHS, moving house, going to university. How does this impact continuity of care? Do you have any recommendations for managing/improving this process)*
j. Does your practice have a protocol for supporting **transition** to AMHS for people with ADHD? (*Do you have experiences of supporting transitions for other LTC that could inform ADHD transitions?)*
k. What are your experiences of communicating with the patient's **parent/carer** when providing support for ADHD? *(transition, involving in care, contacting, help attending appointments, privacy)*
l. Do you have anything to add?

4. Providing care and support for wider mental and physical health through primary care to people with ADHD.

This section asks about your experiences of providing for the wider neurodevelopmental, mental, and physical healthcare needs of young people and adults with ADHD.

a. Is there anyone at your practice or in the primary care network (PCN) with a special interest in mental health or neurodevelopmental difference that can provide psychological or social support to someone with ADHD?
b. Please tell us about any additional roles that are in place at your practice or in the PCN that could provide support to a patient with ADHD. *(social prescriber/ pharmacist/mental health worker… What are your experiences in relation to this?)*
c. Are there any things you consider when providing for the wider healthcare needs of patients with ADHD? *(neurodevelopmental difference/ wider mental & physical health)*
d. What do you think are the most important increased health risks associated with having ADHD? *(How relate to ADHD?) Do you or staff at your practice give targeted advice/support on any of these as part of your approach? If so, please describe.*
e. Do you have anything to add?

5. Information resources and digital solutions

This section asks about information resources to help manage the healthcare of patients with ADHD. The focus is on resources you use to aid your practice, and resources you share with patients to help them understand and self-manage their condition.

a. Please tell us about resources you use to help manage the needs of patients with ADHD *(Recognise possible symptoms; make appropriate referrals; prescribe through shared care; support through transition; provide appropriate advice.*

Printed material/ audio or video clips/ online training/ digital apps/ conversations with colleagues – which useful and why?)

b. Please tell us about resources you signpost patients with ADHD to. *(Which useful and why? Consider self-management, accessing healthcare for ADHD & wider health needs)*

c. In an ideal world, what kind of resources would you like to be available in primary care to help manage ADHD patient needs? *(For patient /clinician use. Local/ national/ digital/ formats. NHS website, video stories, clinical decision-making tools, apps)*
If someone told you about a fab digital tool to aid treating patients with ADHD, what would it be like and what would it do?

d. Please tell us about tools for managing other conditions that could be adapted for use with ADHD (*e.g., clinical decision apps. For clinician/patient use)*

e. Do you have anything to add?

6. Reflection/Close

This section is asking about the advice you would give people who either **provide** or who **need** healthcare for ADHD about how to access care in the current system.

a. What advice would you give to someone working primary care about how to support a young person with ADHD? *(effective treatment & support/ information & resources/ questions to ask & ways of listening)*

b. What advice would you give to a young person with ADHD and their family about how to get help and support for their ADHD through primary care? *(how to access/ ADHD medication/ who to talk to/ what to say / questions to ask)*

c. Is there anything you would like to add?

Interview Topic Guide – WP2
(Young people and parents/carers)

Study title: Managing young people with ADHD in primary care (MAP) study
Chief Investigator: Dr Anna Price a.price@exeter.ac.uk. Telephone 01392 726026

Please note this topic guide is for researchers only. This will be used to prompt questions during the interview. This is not a questionnaire. This topic guide will be adapted and refined following findings from WP1, and in consultation with MAP study research advisory groups.

This interview aims to explore your perspectives and experiences in relation to accessing services and support for young people (aged 16-25) and adults with attention deficit hyperactivity disorder (ADHD) through a primary care provider (e.g., GP practice or local surgery). There will be a particular focus on factors that have made it easier and those that have made it harder to access care. Also, on any recommendations you may have to improve access to primary care for people with ADHD in the future.

Demographics

This section asks for background information about your lived experience of ADHD.

- What is your age (or the age of the person you support)?
- What is the name of your *GP practice? (or postcode)*
- Please tell us briefly about your ADHD diagnosis (or that of the person you support) and any other diagnoses. *(e.g., NHS/Private, age diagnosed, any other diagnoses)*
- What do you (or does the person you support) do? *(training, student, job role; not in education, employment, or training (NEET), prefer not to say)*
- What is your sex (gender)? *(male, female, non-binary / third gender, prefer not to say)*
- Which ethnic group best describes you?
    - *a. Asian, Asian British, Asian Welsh*
    - *b. Black, Black British, Black Welsh, Caribbean or African*
    - *c. Mixed or Multiple*
    - *d. Whiteethnic group*
    - *e. Prefer not to say*

Research topic guide

7. How people with ADHD access primary care

This section asks about your experiences of the ways young people and adults with ADHD make and attend healthcare appointments at their GP practice.

- *a.* How do you (or does the person you support) **make and attend appointments** with your GP practice? What are the barriers? What makes it easier? *(Moving home/ University/ Covid-19/ Face-to-face/online/phone)*
- b. What role does your parent/carer have in helping you to access care and support? (Or what role do you have as a parent/carer, and what role does the young person take)
- c. Is there anything your practice could do to make it easier?

8. Healthcare and support for ADHD through primary care

This section asks about your experiences of healthcare and support for ADHD from your GP, including barriers faced and what makes it easier. We are also interested in differences of experience between NHS and private healthcare routes.

a. What are your experiences of **referral** for a diagnosis of ADHD via your GP? *(private/NHS. How well AMHS & GP communicate)*
b. Please tell us briefly about your **diagnosis** of ADHD (or that of the person you support) and about how this was received. *(NHS/right to choose/private. If private, why?)*

Prescribing

c. Do you (or does the person you support) need medication for your ADHD? *(if not skip questions d & e)*
d. What are your experiences of getting **prescriptions** for ADHD medication from your GP? *(Private/NHS routes. What works/does not work. How AMHS & GP communicate)*
e. Please tell us about your experiences of any **regular checks/review** your GP carries out as part of prescribing ADHD medications. *(Private/NHS)*

Other ADHD healthcare (not medications)

a. Which health professionals have you received ADHD support for at your practice? *(GP/ community worker/ pharmacist/ mental health support worker)*
b. Other than medication, please tell us about **any healthcare** for ADHD you (or the person you support) have received from your local GP practice *(Social prescribing/mental health/support groups). What other healthcare would you find useful?*
c. What are your expectations and needs from a consultation, or other support in primary care for ADHD?
d. What have been your experiences of ADHD healthcare from your GP at times of change in your life? *(transition to AMHS, moving house, going to university. How did this impact continuity of care? Do you have any recommendations for managing/improving this process)*
e. What are your experiences of your practice working together with your supporter (family/friend/partner) to help you get healthcare for ADHD? *(transition, involving in care, contacting, help attending appointments, privacy)*
f. Do you have anything to add?

9. Healthcare and support for wider health needs through primary care

This section asks about your experiences of healthcare and support from your GP for wider physical and mental health needs (when you have ADHD).

a. Do you (or does the person your support) have any co-existing **health** challenges? *(mental health/neurodevelopmental difference/physical)*
b. Have you had a chance to discuss your health more widely with your primary care provider? *(mental health/neurodevelopmental difference/physical)*
c. Are you aware of any **health risks** faced by people with ADHD? (*Have you had a chance to discuss this /received advice? Awareness re substance use, smoking, other risk-taking behaviours; any signposting to support; harm reduction strategies etc.)*
d. What do you feel your practice could do to help you to get your **wider health needs** met when you have ADHD?

e. Do you have anything to add?

10. **Information and resources and digital solutions to help manage ADHD.**
This section asks about resources to provide information to help manage and self-mange the health of people with ADHD.

a. Please tell us about ADHD healthcare resources you have been given by your practice *(printed/ audio or video clips/ online training/ digital apps/ support groups – which useful and why?)*
b. What other resources do you use to help manage health and wellbeing with ADHD? (*which useful, why)*
c. In an ideal world, what resources would be available from your GP to help manage ADHD? *(local, national, digital... What is your preferred method for gaining information about treatment and support for adult ADHD and why?)*
f. If someone told you about a fab digital tool for people with ADHD, what would it be like and what would it do?
d. Please tell us about tools for managing other health conditions which could be adapted for ADHD *(e.g., diabetes or depression health apps, alarms, exercise programmes)*
e. Do you have anything to add?

11. **Reflection/Close**
This section is asking about the advice you would give people who either **provide** or who **need** healthcare for ADHD about how to access care in the current system.

a. What advice would you give to someone working primary care about how to support a young person with ADHD? *(treatment & support/ information & resources /questions to ask /ways of listening)*
b. What advice would you give to a young person with ADHD and their family about how to get help and support for their ADHD through primary care? *(how to access/ ADHD medication/ who to talk to/ what to say / questions to ask)*
c. Is there anything you would like to add?

Table S1 with summary of research site characteristics

Table S1. Sample: overview of research sites, participants, local area profiles, and site descriptions.

Practice research site (number of participants)	Profile of local authority			Profile of practice, including characteristics of local area as described by participants	
	% 'White'[1]	Rural/Urban classification[2]	IMD rank[3]	Summary	Quotes
S1 (n=3)	94	Urban with City and Town	50	~12,500 patients. High deprivation area. Specialists in substance misuse. Contract for homeless outreach. Social prescriber, Mental Health (MH) worker	*"most deprived practice in the city…top 10% of most deprived practices in the country. We see a huge amount of mental health, substance misuse. We're specialists in substance misuse ... We hold the contract for the Outreach to the Homeless Service, and we take our care out to hostels."* *"…we have a social prescriber through the Additional Role Reimbursement Scheme, with the PCNs…that works in our practice…. We've got a mental health worker"* **Practice manager**
S2 (n=5)	84	Urban with Major Conurbation	1	~3,500 patients. High deprivation area. Provide drug misuse services and SAS services. Counsellor.	*"…one of the areas of England with the highest index of multiple deprivation…."* *"inequalities … massive… lack of job opportunities, education, housing, you name it. … high asylum seeker community … a really, really, diverse area."* *"We provide a drug misuse service… SAS service…for patients who have been removed from their practice having behaved in a way that warranted the police being called."* "We have an onsite counsellor who's available for brief interventions" **General practitioner (GP)**
S3 (n=4)	97.6	Largely Rural	75	~7,000 patients. Link workers, MH workers.	"we have link workers, and … primary mental health workers". **GP**
S4 (n=4)	70.7	Urban with City and Town	180	~7,000 patients. University linked practice. High numbers of students and patients from overseas. Wellbeing worker.	*"… we have a young population… We also have a lot of overseas families and students." Practice manager* *"We have a …[wellbeing] worker who comes to the practice who doesn't provide therapy as such but is very good for exploring complex issues and kind of way finding and thinking about other resources that may be needed or accessible to a particular patient."* **GP**
S5 (n=4)	48.6	Urban with Major Conurbation	7	~5,500 patients. High deprivation area. MH worker, social prescriber.	"high deprivation area, …one of the most deprived communities in … in England, so lowest 10%." **GP** "…we have a mental health worker… social prescribers" **Practice manager**

[1]Census 2021, % of people classing themselves as 'White', with average in England being 81.7% (1); [2]Census 2011 (2)); [3]Indices of Multiple Deprivation (IMD) summaries for Local Authority Districts (3), Rank of proportion of LSOAs in most deprived 10% nationally with 1 being highest rank for deprivation and 195 being lowest.

**Published in linked paper (4)*

References

1. Office for National Statistics. Census profile for England and Wales 2021 [cited 2023 31st August]. Available from: https://www.nomisweb.co.uk/sources/census_2021/report.
2. Department for Environment FaRA. 2011 Rural Urban Classification lookup tables for all geographies 2021 [cited 2023 August 31st]. Available from: https://www.gov.uk/government/statistics/2011-rural-urban-classification-lookup-tables-for-all-geographies.
3. Ministry of Housing CLG. English indices of deprivation 2019 2019 [cited 2023 August 31st]. Available from: https://www.gov.uk/government/statistics/english-indices-of-deprivation-2019.
4. Gudka R, Becker K, Ward J, Smith JR, Mughal F, Melendez-Torres GJ, et al. Primary care provision for young people with ADHD: A multi-perspective qualitative study. Br J Gen Pract. 2024;74:e404-e16.
